# Unique branching pattern of aortic arch in a patient with aortopulmonary window

**DOI:** 10.1259/bjrcr.20150298

**Published:** 2016-02-25

**Authors:** Venkatraman Bhat, Karthik Gadabanahalli, Rakesh Sharma, Sejal Shah

**Affiliations:** Department of Radiology and Pediatric Cardiology, Narayana Health, Bangalore, India

## Abstract

Aortic arch (AA) anomalies are usually associated with congenital heart disease. Variations such as aberrant subclavian artery have significance if shunt surgery is planned. Other variations may be clinically insignificant or present with respiratory or oesophageal symptoms. Demonstration and understanding of arch anomalies are crucial for managing as well as improving our understanding of their embryological basis. This presentation illustrates an unusual branching pattern of AA in a patient with an aortopulmonary window in which five arteries independently arose from the AA. CT imaging appearance of the anomaly is illustrated. A brief description of the embryological basis and significance of the anomaly is presented.

## Case report

**Figure 1.**
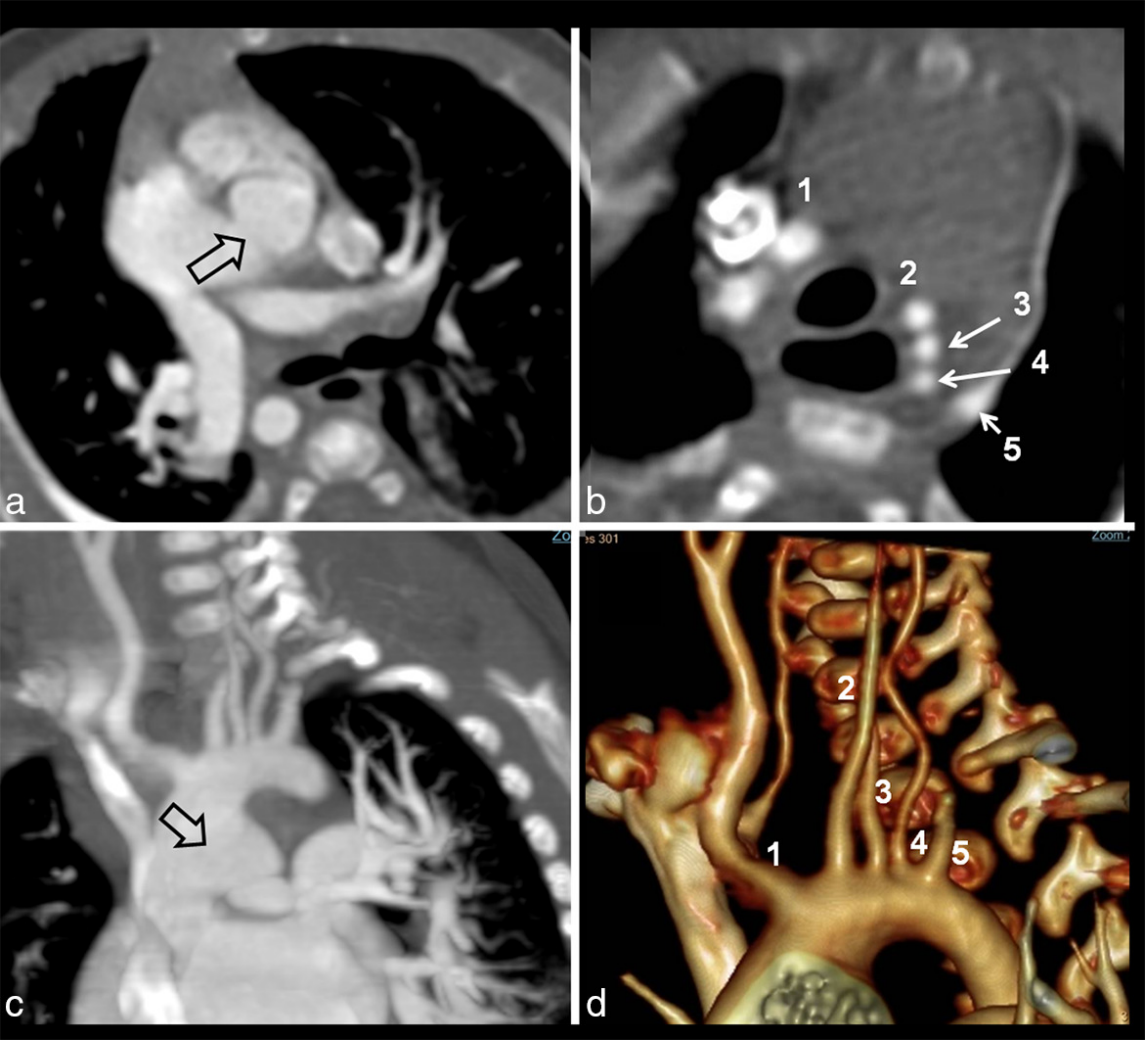
(a) Axial CT image at the level of main pulmonary artery shows an APW (open arrow). Axial image above the level of AA (b) shows six arteries, five directly arising from the aorta (1, innominate artery; 2, LECA; 3, left ICA; 4, left VA; and 5, LSA). Coronal maximum intensity projection image (c) demonstrates an APW (open arrow) and five arteries arising from the AA. Three-dimensional surface rendered view (d) showing the branches of AA (1–5). AA, aortic arch; APW, aortopulmonary window; ICA, internal carotid artery; LECA, left external carotid artery; LSA, left subclavian artery; VA, vertebral artery.

A 27-day-old infant, who presented with failure to thrive, was incidentally detected to have a cardiac murmur. The infant was born preterm and was small for gestational age. There was no cyanosis. Clinical examination was unremarkable except for a systolic ejection murmur in the precordial region. Echocardiography revealed the presence of a large aortopulmonary window (APW) and a 7-mm secundum atrial septal defect. There was bidirectional shunting across the APW with evidence of severe pulmonary arterial hypertension. The patient was investigated further with a CT scan for assessment of the additional cardiac anomalies. A multidetector CT evaluation revealed large Type 1 APW (Figure 1a,c). Additionally, the aortic arch (AA) showed a unique branching pattern characterized by separate origins of the brachiocephalic artery (BCA), left external carotid artery (LECA), left internal carotid artery (ICA), left vertebral artery (VA) and left subclavian artery (LSA); overall, a five-vessel configuration of the AA (Figure 1b–d) was seen. The patient was offered surgical correction of the APW. No intervention was considered necessary for the AA anomaly.

## Discussion

Routine investigations of congenital heart disease with non-invasive imaging methods have broadened our understanding about the incidence and complexity of various forms of aortic anomalies.^1^ Typically, the AA is left-sided and shows a branching pattern of the three great vessels, starting with the BCA, left common carotid artery (LCCA) and LSA from right to left. This classical branching pattern occurs in 64.9–94.3% of the cases.^1,2^ Common variations of the AA are the two-branch configuration, also called the bovine arch (BCA with LCCA and LSA), showing an incidence of 10–22%;^1^ direct origin of the left VA (incidence 2.4–8%) arising either between the LCCA and LSA or as a last branch; aberrant LSA or right subclavian artery (RSA; incidence 1%); and direct origin of both VAs from the AA. Anomalies of arch arteries are also associated with double AA. Edwards^3^ proposed an embryological concept of development of AAs which can provide a basis for understanding anomalies. Natsis et al^4^ proposed a classification of the AA vessels into eight categories based on a digital subtraction angiographic study. Type I refers to a “normal” AA branching into the BCA, LCCA and LSA. In Type II, the LCCA originates from the BCA. In Type III, the left VA directly leaves the AA between the LCCA and the LSA. In Type IV, the common carotid arteries (CCAs) originate from the common trunk arising between the SAs. In Type V, the CCAs originate from a common trunk and a right aberrant SA is present. In Type VI, the CCAs and the SAs originate from individual common trunks. In Type VII, the BCA is absent and the RSA, right CCA, LCCA and LSA leave the AA separately. In Type VIII, thyroid inferior mesenteric artery originates from the AA.

**Figure 2.**
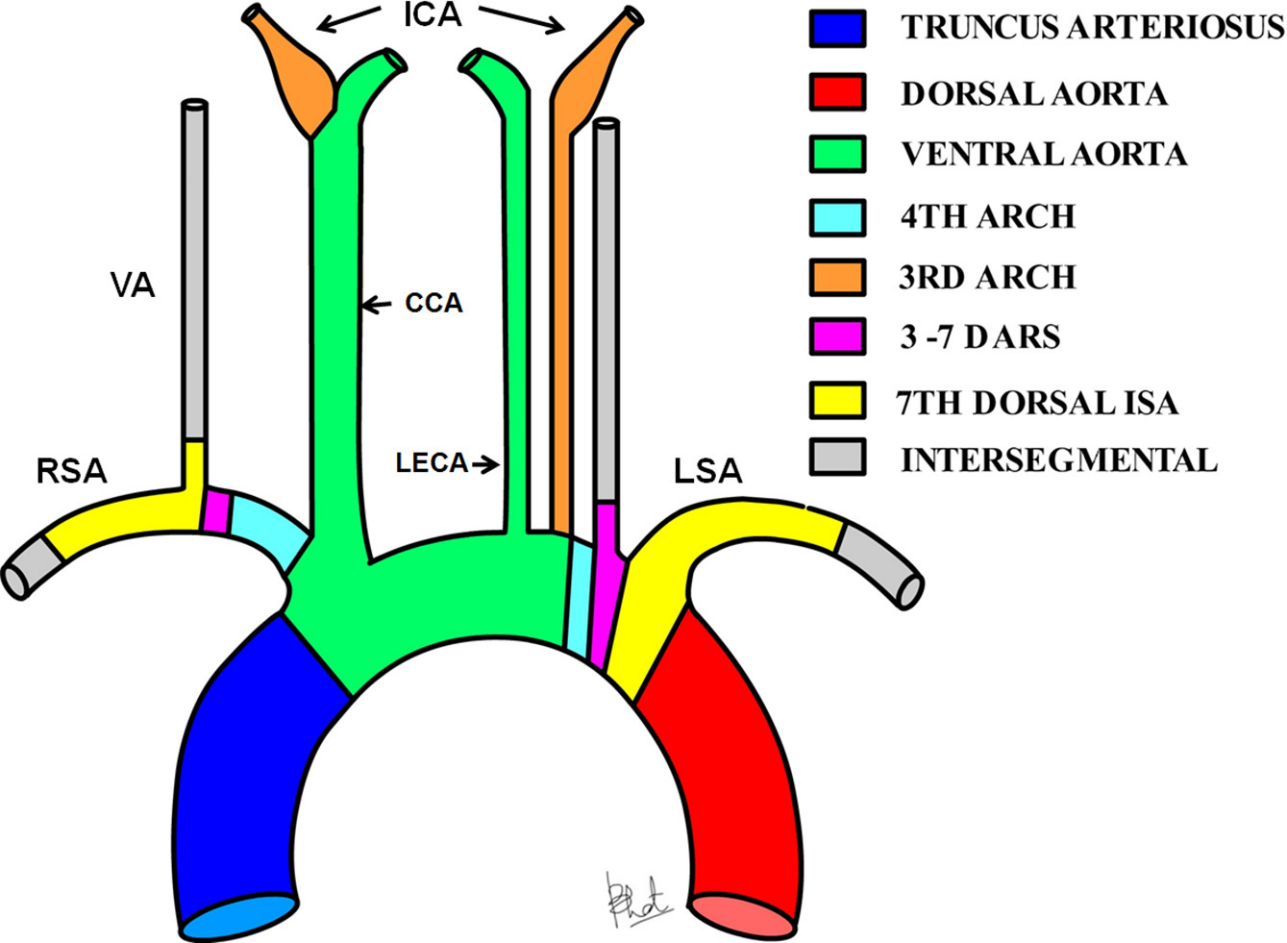
The diagram illustrates the likely embryological basis of direct origins of the LECA and left ICA, and the left VA (modified from Satti et al^8^ with permission from the American Society of Neuroradiology). CCA, common carotid artery; DARS, dorsal aortic root segments; DISA, dorsal intersegmental artery; ICA, internal carotid artery; LECA, Left external carotid artery; LSA, left subclavian artery; RSA, right subclavian artery; VA, vertebral artery.

Our case does not belong to any of the previously mentioned categories. A review of the literature indicates few cases of separate origins of the LECA and the left ICA.^5–7^ No instances are available reporting a direct origin of the left ICA, LECA and VA from the AA in association with an APW. Embryological basis of some anomalies are suggested. The persistence of first or second dorsal intersegmental artery connecting to the fourth left AA presumably leads to the direct origin of the left VA from the AA.^8^ The ICA originates from the third dorsal arch; caudal migration of the point of union with the ventral arch can lead to direct aortic origin (Figure 2). Our unique example adds to the knowledge of the spectrum of AA anomaly.^7^ Knowledge of the unusual anatomical variation is very valuable to an interventional radiologist for selective study of the neck vessels.

## Learning points

1. AA anomalies are common in association with congenital heart disease; some anomalies have clinical significance.
2. A large majority (64.9–94.3%) of AAs have a three-vessel configuration consisting of BCA, LCCA and LSA.
3. Independent origin of more than four arteries is very unusual. Direct origin of the LECA and left ICA is an anatomic and embryological curiosity.
4. Documentation of aortic anomalies are helpful for selective catheterization of neck arteries for diagnostic or interventional purposes.
